# Association between obesity and new-onset heart failure among patients with hypertension in Thailand

**DOI:** 10.1186/s41043-024-00530-6

**Published:** 2024-02-29

**Authors:** Boonsub Sakboonyarat, Jaturon Poovieng, Ram Rangsin

**Affiliations:** 1 Department of Military and Community Medicine, Phramongkutklao College of Medicine, Bangkok, 10400 Thailand; 2 Pulmonary and Critical Care Division, Department of Medicine, Phramongkutklao College of Medicine, Bangkok, 10400 Thailand

**Keywords:** Heart failure, Obesity, Body mass index, Hypertension, Thailand

## Abstract

**Background:**

In Thailand, the epidemiological data on the relationship between obesity and heart failure (HF) among high-risk populations was limited. We assessed the association between body mass index (BMI) and the new-onset HF among people with hypertension (HTN), and also assessed the effect modifier of uncontrolled HTN on this association.

**Methods:**

We analyzed the data obtained from the 2018 Thailand DM/HT study database. Thai people with HTN aged 20 years and older receiving continuous care at outpatient clinics in hospitals nationwide were included. The new-onset HF was defined regarding the ICD-10 as I50 in the medical records within 12 months. Obesity was defined as BMI $\ge$ 25 kg/m^2^. Multivariable log-binomial regression analysis was used to determine the association between BMI and new-onset HF and presented as the adjusted risk ratio (aRR) and 95% confidence interval (CI).

**Results:**

A total of 35,756 participants were included in the analysis. In all, 50.0% of the participants had BP control for the last two consecutive visits. The mean BMI was 25.1 $\pm$ 4.7 kg/m^2^. New-onset HF occurred in 75 participants (0.21%; 95% CI 0.17–0.26). After adjusting for potential confounders, an elevated BMI was associated with new-onset HF (*p* value for quadratic trend < 0.001). In comparison with participants with normal BMI (18.5–22.9 kg/m^2^), the aRR for new-onset HF was 1.57 (95% CI 0.80–3.07) and 3.97 (95% CI 1.95–8.10) in those with BMI 25.0–29.9, and ≥ 30.0 kg/m^2^. For participants with obesity, aRR for new-onset HF was 2.05 (95% CI 1.24–3.39) compared to non-obese participants. The study found that among patients with control BP, obesity was associated with a higher risk of new-onset HF with an adjusted RR of 2.33 (95% CI 1.12–4.83). For those with uncontrolled BP, the adjusted RR was 1.83 (95% CI 0.93–3.58), but there was no heterogeneity with *p* value = 0.642.

**Conclusion:**

An increased BMI had a higher risk for new-onset HF among Thai people with HTN. Obesity was independently associated with new-onset HF among people with HTN, regardless of uncontrolled HTN. Our findings highlight that weight reduction is crucial for mitigating the risk of HF development in HTN patients, regardless of their BP control status.

**Supplementary Information:**

The online version contains supplementary material available at 10.1186/s41043-024-00530-6.

## Background

Heart Failure (HF) is a complex and life-threatening syndrome that poses a significant public health problem worldwide [1, 2]. In 2017, the number of individuals affected by HF was estimated to be over 64 million globally [2–4]. Although the incidence of HF has stabilized at approximately 1–20 cases per 1,000 person-years in developed countries, its mortality rate remains high, with a 1-year mortality rate of approximately 25–30% [3, 4]. Robust evidence indicates that HF is a leading cause of morbidity and mortality, particularly in high-risk populations such as those with hypertension (HTN) [2, 3].

Overweight and obesity are considered global health concerns and are known metabolic risk factors for noncommunicable diseases, particularly cardiovascular disease (CVD) [5, 6]. Previous studies have shown that an increase in body mass index (BMI) is associated with a higher risk of HF [7–9]. For instance, a 1 kg/m^2^ increase in BMI was linked to an 11% increase in the risk of HF, according to a large-scale prospective cohort study of the Physicians' Health Study [9]. Similarly, the Framingham Heart Study reported that individuals with BMI in the range of 25.0–29.9 kg/m^2^ had an adjusted hazard ratio of 1.5 (95% CI 1.3–1.7) for the risk of HF compared to those with BMI < 25 kg/m^2^ [8]. However, a reverse epidemiology was observed among Hispanic males in the Multi-Ethnic Study of Atherosclerosis [7]. Another study conducted in the Asian population found higher HF hospitalization rates in individuals with low BMI [10].

In Thailand, the prevalence of metabolic risk factors for CVD, including HTN and obesity, has been on the rise [11–15]. Approximately one-fourth of Thai adults are affected by HTN, and approximately two-thirds (66.6%) of Thai patients with HTN can control their blood pressure (BP) [16]. The prevalence of obesity (BMI ≥ 25 kg/m^2^) among Thai adults is 37.8% and 46.4% in men and women, respectively [11]. A recent study, the Thailand DM/HT study, reported that almost half of Thai patients with HTN (47.6%) have obesity [16].

According to existing evidence in Thailand, approximately 1% of patients with HTN have a history of HF, and the prevalence of HF increases significantly with age, particularly in individuals older than 65 years [16]. However, epidemiological data on the relationship between obesity and HF among high-risk populations, particularly those with HTN, remain limited in Thailand. Therefore, in the present study, we aimed to assess the association between increased BMI and new-onset HF among patients with HTN receiving continuous care in Thailand, as well as to evaluate the effect modifier of BP control status on this association.

## Methods

### Study designs and subjects

In the present study, we obtained the data from the Thailand DM/HT study database in 2018, supported by the National Health Security Office (NHSO). As described elsewhere by Sakboonyarat et al.[16], the Thailand DM/HT study, an annual survey, focused on evaluating clinical outcomes, such as BP control and cardiovascular complications, in patients with HTN aged 20 and above who received HTN care for at least 12 months at outpatient clinics in hospitals across the country. However, the study did not include patients who had a history of pregnancy within 12 months from the date of data collection and patients receiving care at university hospitals.[16]

The study database on DM/HT in Thailand during the period of January 2018 to July 2018 comprised an initial sample size of 36,557 hypertensive patients [16]. After excluding 530 subjects without BMI data in their medical records and 271 individuals with a history of HF prior to 12 months from the date of data collection (i.e., before January 2017 for the data collection date on January 2018), a total of 35,756 hypertensive patients without baseline HF and having information on BMI were included in the final analysis. Regarding the retrospectively collected database, with a short follow-up period, we examined the cross-sectional association between obesity and the risk of new-onset HF.

### Data collection

In the Thailand DM/HT study, the registered nurse, who was trained, meticulously reviewed and abstracted the data from medical records using a case report form (CRF) that was based on a standardized protocol. Once the data was carefully collected, it was then transmitted to the data management unit located in Nonthaburi province [16, 17]. The information that was collected included a comprehensive analysis of the demographic characteristics of patients with HTN, health insurance scheme, the location of the outpatient clinic, weight and height, smoking status, alcohol use, comorbidities, the history of antihypertensive medication use, BP, and any cardiovascular complications such as ischemic heart disease (IHD) and HF.

### Outcome and exposure

In the present study, the new-onset HF was defined as the occurrence of HF as a new onset in hypertensive patients within 12 months until the data collection date. The definition was based on the International Classification of Diseases, Tenth Revision Codes (ICD-10) I50, which appeared in patients' medical records [18]. It is important to note that in Thailand, patients are covered by health insurance schemes, such as Universal Health Coverage (UHC), Civil Servant Medical Benefit (CSMB), or Social Security (SS). As such, when a patient develops new-onset HF, they are typically able to receive appropriate and standardized care based on their specific health insurance coverage. Regarding the exposure of interest, BMI was calculated based on the recorded weight and height of the subjects during their most recent medical visit. The BMI values were segregated into five categories: 18.5–22.9 kg/m^2^ (normal weight), < 18.5 kg/m^2^ (underweight), 23.0–24.9 kg/m^2^ (overweight), 25.0–29.9 kg/m^2^ (obese I), and ≥ 30 kg/m^2^ (obese II). Furthermore, obesity was defined as BMI equal to or greater than 25 kg/m^2^.[19]

### Covariates

The covariates considered in this study comprised a range of demographic variables, including age and sex, as well as a variety of health-related information. Geographic regions were also taken into account, with North, Central, Northeast, and South all being included. In terms of health insurance schemes, UHC, CSMB, SS and others were all encompassed. Hospital location of the clinic was categorized into regional hospital, general hospital, community hospital, private hospital, and others, while comorbidities were defined based on the ICD-10, including type 2 diabetes (T2D) (E11), dyslipidemia (DLP) (E78), and renal insufficiency (N18) [18]. History of IHD was defined based on the ICD-10: I20-I25 [18], or a recorded history of coronary revascularization [20]. Tobacco use was determined based on the smoking status recorded in the medical records as either never smoked or ever smoked, and alcohol consumption was similarly determined based on the history of alcohol use recorded in the medical records as either never alcohol use or ever alcohol use. Angiotensin-converting enzyme inhibitors or angiotensin receptor blockers (ACEI/ARB) use was defined as a history of ACEI/ARB being prescribed in the medical record within 12 months before the data collection date. BP control was defined as systolic BP < 140 mmHg and diastolic BP < 90 mmHg, and BP control for two consecutive times was defined as BP control at both the latest visit and one time before the latest visit [16, 21].

### Statistical analysis

The present study employed StataCorp. 2021. *Stata Statistical Software: Release 17*. College Station, TX: StataCorp LLC, for all data analysis. Descriptive statistics was utilized to characterize the study participants, where categorical data was presented in percentages, while continuous data was presented as mean with standard deviation (SD) and median with Q1 and Q3. A *chi*-square test was used to compare the characteristics of participants between the obesity and non-obesity groups for categorical variables, while a *t*-test was employed for continuous variables. To evaluate the association between BMI and new-onset HF, a log-binomial regression analysis was conducted and presented as a risk ratio (RR) and 95% confidence interval (CI). The multivariable analysis adjusted for several covariates, including model 1: age, and sex; model 2: variables in model 1 plus health insurance schemes, geographic regions, location of clinic, T2D, DLP, renal insufficiency, history of IHD, smoking status, alcohol use, control BP consecutively two latest visits, and ACEI/ARB use, and presented as adjusted RR, and 95% CI. The average adjusted prediction of new-onset HF by increased BMI was illustrated using the margins command.

Furthermore, a subgroup analysis was performed using multivariable log-binomial regression analysis to evaluate the association between obesity and the risk of new-onset HF among patients with control BP for the last two consecutive visits and among those with uncontrolled BP. The interaction was tested to explore whether BP control status modifies the association between obesity and new-onset HF. A statistical significance was considered by a two-sided *p* value less than 0.05.

### Sensitivity analysis

Despite adjusting for potential confounders in the multivariable model in the primary analysis, we did not have the opportunity to include physical activity and dietary behavior in the final model, which means that residual confounding may exist. Therefore, we conducted a sensitivity analysis for unmeasured confounding using E-values estimated by the evalue package [22].

### Ethic considerations

The study was reviewed and approved by the Institutional Review Board, the Royal Thai Army Medical Department, in compliance with international guidelines such as the Declaration of Helsinki, the Belmont Report, CIOMS Guidelines, and ICH-GCP (approval number S055h/65_Exp). A waiver of documentation of informed consent was utilized due to the use of secondary data, and was granted by the Institutional Review Board, the Royal Thai Army Medical Department.

## Results

### Characteristics of study participants

The present study included a total of 35,756 patients with HTN who received continuous care. The majority of participants (61.4%) were women, and the average age of participants was 64.5 ± 11.8 years. Of all the participants, 74% were under the UHC scheme. Regarding comorbidities, 13.1%, 15.4%, and 62.8% of the study participants had a history of renal insufficiency, T2D, and DLP, respectively. Our analysis revealed that 50.0% of the participants had BP control for the last two consecutive visits. The average BMI of the participants was 25.1 ± 4.7 kg/m^2^. The prevalence of obesity (BMI ≥ 25 kg/m^2^) was found to be 47.1%. Table 1 presents the characteristics of the participants in the study and stratified by obesity.

**Table 1:** Characteristics of study participants (N = 35,756)

Characteristics	Total	BMI < 25 kg/m^2^	BMI $\geq$ 25 kg/m^2^	*p*-value
	n (%)	n (%)	n (%)	
Total N	35,756	18,901 (52.9)	16,855 (47.1)	
Sex				< 0.001
Women	21,950 (61.4)	10,974 (58.1)	10,976 (65.1)	
Men	13,806 (38.6)	7927 (41.9)	5879 (34.9)	
Age, years				< 0.001
Mean (SD)	64.5 (11.8)	67.8 (11.5)	60.9 (11.0)	
Median (Q1–Q3)	64.0 (56.0–73.0)	68.0 (60.0–76.0)	61.0 (53.0–68.0)	
Regions				< 0.001
North	9736 (27.2)	5402 (28.6)	4334 (25.7)	
Central	10,057 (28.1)	5007 (26.5)	5050 (30.0)	
Northeast	9877 (27.6)	5285 (28.0)	4592 (27.2)	
South	6086 (17.0)	3207 (17.0)	2879 (17.1)	
Location of outpatient clinics				< 0.001
Regional Hospital	2305 (6.5)	1073 (5.7)	1232 (7.3)	
General Hospital	5747 (16.1)	2842 (15.0)	2905 (17.2)	
Community Hospital	25,019 (70.0)	13,769 (72.9)	11,250 (66.8)	
Private Hospital	435 (1.2)	199 (1.1)	236 (1.4)	
Others	2250 (6.3)	1018 (5.4)	1232 (7.3)	
Health insurance scheme				< 0.001
Universal health coverage	26,459 (74.0)	14,250 (75.4)	12,209 (72.4)	
Civil servant medical benefit	7107 (19.9)	3770 (20.0)	3337 (19.8)	
Social security	1581 (4.4)	561 (3.0)	1020 (6.1)	
Others	609 (1.7)	320 (1.7)	289 (1.7)	
Smoking status				< 0.001
Never	29,048 (83.2)	14,966 (80.9)	14,082 (85.8)	
Ever smoker	5868 (16.8)	3531 (19.1)	2337 (14.2)	
Alcohol use				< 0.001
Never	4118 (11.7)	2394 (12.8)	1724 (10.4)	
Ever alcohol use	31,226 (88.4)	16,324 (87.2)	14,902 (89.6)	
Type 2 diabetes				< 0.001
No	30,269 (84.7)	16,433 (86.9)	13,836 (82.1)	
Yes	5487 (15.4)	2468 (13.1)	3019 (17.9)	
History of ischemic heart disease				< 0.001
No	34,536 (96.6)	18,169 (96.1)	16,367 (97.1)	
Yes	1220 (3.4)	732 (3.9)	488 (2.9)	
History of dyslipidemia				< 0.001
No	13,291 (37.2)	7654 (40.5)	5637 (33.4)	
Yes	22,465 (62.8)	11,247 (59.5)	11,218 (66.6)	
History of renal insufficiency				< 0.001
No	31,088 (86.9)	15,863 (83.9)	15,225 (90.3)	
Yes	4668 (13.1)	3038 (16.1)	1630 (9.7)	
History of ACEI/ARB use				< 0.001
No	14,008 (39.2)	8123 (43.0)	5885 (34.9)	
Yes	21,748 (60.8)	10,778 (57.0)	10,970 (65.1)	
BP control for the latest two consecutive visits				< 0.001
No	17,671 (50.0)	8913 (47.8)	8758 (52.5)	
Yes	17,663 (50.0)	9749 (52.2)	7914 (47.5)	
Body mass index (kg/m^2^)				< 0.001
Mean (SD)	25.1 (4.7)	21.7 (2.4)	28.9 (3.5)	
Meadina (Q1–Q3)	24.7 (22.0–27.8)	22.0 (20.1–23.6)	28.0 (26.4–30.4)	

*BMI* body mass index, *ACEI/ARB* angiotensin-converting enzyme inhibitors/angiotensin receptor blockers, *BP* blood pressure, *SD* standard deviation

### Association between obesity and new-onset heart failure

**Fig. 1:**
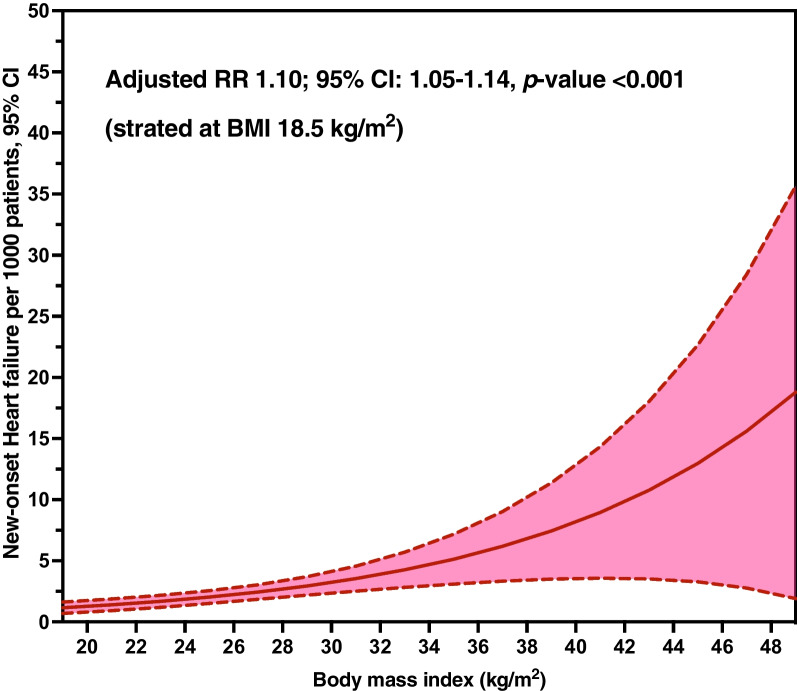
Average adjusted prediction of risk of new-onset heart failure per 1000 patients with hypertension and 95% CI. RR: relative risk, CI: confidence interval. Average adjusted prediction of risk of new-onset heart failure per 1000 patients and 95% CI per 1 kg/m^2^ of BMI (started at 18.5 kg/m^2^), adjusting for age, sex, health schemes, geographic regions, hospital types, type 2 diabetes, dyslipidemia, renal insufficiency, history of ischemic heart disease, smoking status, alcohol use, control blood pressure consecutively two latest visits, and ACEI/ARB use.

Within a year, 75 new-onset HF events were observed, accounting for 0.21% (95% CI 0.17–0.26) of the study population. Univariable log-binomial regression analysis for factors associated with new-onset HF is presented in Additional file 1: Table S1. The association between BMI and new-onset HF was analyzed through a multivariable log-binomial regression, as presented in Table 2. After adjusting for potential confounders, the analysis revealed that an elevated BMI was associated with new-onset HF (*p* value for quadratic trend < 0.001). Figure 1 demonstrates the increased BMI (started at 18.5 kg/m^2^) for the average adjusted prediction of new-onset HF per 1000 patients and 95% CI. Further, the high categories of BMI, i.e., 25.0–29.9 kg/m^2^ and ≥ 30 kg/m^2^, were associated with an increased risk of new-onset HF, as compared to the normal BMI category (18.5–22.9 kg/m^2^), with adjusted RRs of 1.57 (95% CI 0.80–3.07) and 3.97 (95% CI 1.95–8.10), respectively. Among the overall participants, individuals with obesity (BMI ≥ 25 kg/m^2^) had a higher risk of new-onset HF (adjusted RR 2.05; 95% CI 1.24–3.39), as compared to those with non-obesity (BMI < 25 kg/m^2^). Similarly, the adjusted RR for new-onset HF in HTN patients with BMI ≥ 30.0 kg/m^2^ was 3.19 (95% CI 1.85–5.57) compared to those with BMI < 30 kg/m^2^. Table 3 presents the association between BMI and new-onset HF stratified by BP control for the last two consecutive visits. Among patients with control BP, the adjusted RR for the association between obesity (BMI ≥ 25 kg/m^2^) and new-onset HF was 2.33 (95% CI 1.12–4.83), while the adjusted RR was insinuated as 1.83 (95% CI 0.93–3.58) among those with uncontrol BP. However, the interaction testing revealed no heterogeneity with *p *value = 0.642.

**Table 2:** Univariable and multivariable log-binomial regression for the association between body mass index and new-onset of heart failure among Thai patients with hypertension

Variables	Total	New-onset HF	Univariable		Multivariable Model I*		Multivariable Model II**	
	N	n (%)	cRR (95% CI)	*p-v*alue	aRR (95% CI)	*p-v*alue	aRR (95% CI)	*p-*value
Body mass index (BMI)								
Continuous (linear)	35,756	75 (0.21)	1.06 (1.01–1.10)	0.013	1.10 (1.06–1.15)	< 0.001	1.09 (1.05–1.14)	< 0.001
Continuous (quadratic)	35,756	75 (0.21)	1.001 (1.001–1.002)	0.001	1.001 (1.001–1.002)	< 0.001	1.001 (1.001–1.002)	< 0.001
BMI category, kg/m^2^								
< 18.50	2,248	6 (0.27)	1.54 (0.61–3.90)	0.364	1.10 (0.43–2.82)	0.833	0.98 (0.36–2.69)	0.969
18.50–22.99	9,798	17 (0.17)	Ref		Ref		Ref	
23.00–24.99	6,855	11 (0.16)	0.92 (0.43–1.97)	0.840	1.13 (0.53–2.42)	0.751	1.11 (0.50–2.47)	0.787
25.00–29.99	12,035	21 (0.17)	1.01 (0.53–1.91)	0.986	1.46 (0.76–2.80)	0.262	1.57 (0.80–3.07)	0.186
≥ 30.00	4,820	20 (0.41)	2.39 (1.25–4.56)	0.008	4.21 (2.13–8.32)	< 0.001	3.97 (1.95–8.10)	< 0.001
BMI ≥ 25 kg/m^2^								
No	18,901	34 (0.18)	Ref.		Ref.		Ref.	
Yes	16,855	41 (0.24)	1.35 (0.86–2.13)	0.193	1.96 (1.21–3.17)	0.006	2.05 (1.24–3.39)	0.005
BMI ≥ 30 kg/m^2^								
No	30,936	55 (0.18)	Ref.		Ref.			
Yes	4,820	20 (0.41)	2.33 (1.40–3.89)	0.001	3.49 (2.04–5.96)	< 0.001	3.19 (1.83–5.57)	< 0.001

*BMI* body mass index, *cRR* crude risk ratio, *aRR* adjusted risk ratio, *95% CI* 95% confidence interval

^*^Model I: Adjusting for age and sex

^**^Model II: Adjusting for age, sex, health schemes, geographic regions, location of outpatient clinic, type 2 diabetes, dyslipidemia, renal insufficiency, history of ischemic heart disease, smoking status, alcohol use, control blood pressure consecutively two latest visits, and ACEI/ARB use

**Table 3:** Multivariable log-binomial regression for the association between body mass index and new-onset of heart failure among Thai patients with hypertension, stratified by blood pressure control for the latest two consecutive visits

Variables	Control BP		Uncontrol BP		*p* for interaction
	aRR (95% CI)*	*p-*value	aRR (95% CI)*	*p*-value	
Body mass index (BMI)					
Continuous (linear)	1.10 (1.03–1.17)	0.007	1.09 (1.04–1.14)	< 0.001	0.477
Continuous (quadratic)	1.002 (1.001–1.002)	0.010	1.001 (1.001–1.002)	< 0.001	0.763
BMI category, kg/m^2^					0.602
< 18.50	0.55 (0.12–2.57)	0.447	1.62 (0.41–6.32)	0.490	
18.50–22.99	Ref.		Ref.		
23.00–24.99	1.22 (0.41–3.68)	0.720	1.04 (0.33–3.29)	0.948	
25.00–29.99	1.61 (0.62–4.17)	0.327	1.53 (0.59–3.99)	0.382	
≥ 30.00	4.85 (1.77–13.28)	0.002	3.21 (1.17–8.80)	0.024	
BMI ≥ 25 kg/m^2^					0.642
No	Ref.		Ref.		
Yes	2.33 (1.12–4.83)	0.023	1.83 (0.93–3.58)	0.080	
BMI ≥ 30 kg/m^2^					0.926
No	Ref.		Ref.		
Yes	4.05 (1.73–9.51)	0.001	2.52 (1.25–5.06)	0.010	

*BMI* body mass index, *BP* blood pressure, *aRR* adjusted Risk Ratio, *95% CI* 95% confidence interval

^*^Multivariable analysis: adjusting for age, sex, health schemes, geographic regions, location of outpatient clinic, type 2 diabetes, dyslipidemia, renal insufficiency, history of ischemic heart disease, smoking status, alcohol use, control blood pressure consecutively two latest visits, and ACEI/ARB use

Regarding the sensitivity analysis, the E-value for the RR to identify the association between the unmeasured confounder and the factors associated with new-onset HF is presented in Additional file 1: Table S2. The E-value for the point estimate of the association between obesity and new-onset HF (aRR 2.05; 95% CI 1.24–3.39) was 3.52. therefore, the observed RR of 2.05 could be explained away by an unmeasured confounder that was associated with both obesity and HF by an RR of 3.52-fold each, above and beyond the measured confounders, but weaker confounding could not do so [22].

## Discussion

The results of our study demonstrate a noteworthy and positive association between obesity and the risk of new-onset HF among patients with HTN receiving continuous care in Thailand. Moreover, we identified a quadratic association between elevated BMI and increased risk of new-onset HF in this population. Notably, subgroup analysis stratified by the status BP control for the last two consecutive visits revealed that the association between obesity and new-onset HF was mitigated among those with uncontrolled BP; however, no heterogeneity was observed. It is worth mentioning that this study is the most extensive and up-to-date investigation to date that examines the relationship between BMI and HF risk among patients with HTN who receive continuous care in Thailand. Our findings suggest that obesity is an independent risk factor for new-onset HF in patients with HTN, whether controlling BP and weight reduction may be effective strategies to alleviate the risk of HF in this population.

In the present research, our findings revealed a significant association between BMI and the risk of new-onset HF, with each unit increase in BMI (started at 18.5 kg/m^2^) resulting in a 10% increase in the risk of new-onset HF. Moreover, our investigation disclosed that individuals categorized as overweight, obesity I, and obesity II exhibited an elevated risk of new-onset HF, estimated to be 11%, 57%, and 297%, respectively, compared to those with normal weight. Notably, when assessing the risk of new-onset HF among HTN patients with BMI ≥ 25 kg/m^2^, which is the cutoff for defining obesity status among the Asian population [19], the results demonstrated a relatively precise estimate, with a calculated risk of 2.1 times higher among individuals with BMI ≥ 25 kg/m^2^ compared to those with BMI < 25 kg/m^2^, with a 95% CI of 1.2–3.4. Furthermore, when we use the cutoff at 30 kg/m^2^, we found that the risk of new-onset HF among HTN patients with BMI ≥ 30 kg/m^2^ was 3.2 times the risk of that among those with BMI < 30 kg/m^2^ which indicates the robust of our findings among high-risk population.

The results of our study are consistent with previous research. Notably, a large-scale prospective cohort study of 21,094 men conducted as part of the Physicians' Health Study found that every 1 kg/m^2^ increase in BMI was associated with an 11% increase in the risk of HF [9]. Furthermore, this study showed that individuals with BMI in the range of 25.0–29.9 kg/m^2^ had an adjusted hazard ratio of 1.5 (95% CI 1.3–1.7) for the risk of HF, compared to those with BMI < 25 kg/m^2^ [9]. Similarly, the Framingham Heart Study (FHS) also reported that every 1 kg/m^2^ increase in BMI was linked to a 6% increase in the risk of HF [8]. The FHS further demonstrated that the risk of HF was notably higher among individuals with BMI in the range of 25.0–29.9 kg/m^2^ and ≥ 30 kg/m^2^, compared to those with BMI in the range of 18.5–24.9 kg/m^2^, with adjusted hazard ratios of 1.3 (95% CI 1.1–1.7) and 2.0 (95% CI 1.6–2.6), respectively [8].

On the other hand, in the Multi-Ethnic Study of Atherosclerosis, BMI was found to be positively associated with HF incidence among Caucasians and African Americans. In contrast, a paradoxical or reverse epidemiology between BMI and HF incidence was observed among Hispanic males [7]. In addition, the study in the Asian population also found that the HF hospitalization rate in individuals with high BMI was higher than in those with low BMI [10]. However, the obesity paradox may be an artifact of residual confounding at the statistical level, according to existing literature [23, 24].

In the present study, we observed that the new-onset HF was 2.3 times higher among HTN patients with obesity as compared to those without obesity who had controlled BP. However, among HTN patients with uncontrolled BP, this association between obesity and new-onset HF was alleviated to 1.8. However, no heterogeneity was observed in our study. Based on the solid evidence available, it can be inferred that uncontrolled HTN leads to an increased likelihood of cardiac remodeling, while HF is associated with structural and hemodynamic changes in the heart [25–27]. Thus, it is probable that cardiac remodeling exists in individuals with uncontrolled BP, making them more susceptible to developing HF. This could potentially weaken the association between elevated BMI and new-onset HF, particularly among individuals with uncontrolled BP compared to those with controlled BP. Our findings suggest that weight reduction should be encouraged in HTN patients, whether to control BP or not, to alleviate the risk of HF in the future.

Multiple mechanisms have been proposed to explain the observed independent association between obesity and the development of new-HF. One possible mechanism is the increased metabolic demand resulting from excessive adipose tissue and fat-free mass in obesity, which leads to hyperdynamic circulation, including increased blood volume and stroke volume [28, 29]. These changes can result in hemodynamic overload and increased cardiac stroke work, eventually causing left ventricular failure [28–32]. Another mechanism is the alteration in cardiac structure, which is due to the excessive epicardial fat commonly found in obesity [31, 33]. The epicardial fat, which is strongly associated with visceral adiposity, extends into the myocardium, resulting in fatty infiltration and fibrosis, which can facilitate left ventricular hypertrophy and cardiac dysfunction [29, 32–35]. A third possible mechanism is cardiac lipotoxicity, whereby adiposity promotes ectopic deposition of triglyceride in the heart, leading to cardiac steatosis [36–38]. Cardiomyocytes have limited storage capacity, and excess free fatty acids are shunted into the nonoxidative pathway, leading to lipotoxicity and facilitating apoptosis of lipid-filled cardiomyocytes [29, 37–39]. Additionally, Obesity is often accompanied by comorbidities such as obstructive sleep apnea and obesity hypoventilation syndrome [33]. These conditions increase the demand for ventilation and breathing workload, making respiratory muscles less efficient. Consequently, alveolar hypoventilation and ventilation-perfusion mismatch occur, resulting in pulmonary HTN due to hypoxia-induced vasoconstriction. This can lead to right ventricular failure [30, 40].

The present study has some limitations. One significant limitation is the relatively brief duration of the observation period for the outcome within 12 months. While the findings suggest a positive association between higher BMI and new-onset HF among patients with HTN, the causal inference cannot be drawn from these results. To ensure that the outcome of interest, namely new-onset HF, occurred within 12 months, we excluded HTN patients with a history of HF before 12 months of the data collection date. We abstracted information on the BMI of participants from the latest visit within the 12-month period, which is the same period during which new-onset HF occurs. However, it is important to note that longitudinal studies have documented only slight changes in BMI over a decade in midlife and older adults [41]. In the present study, HF was defined using ICD-10, which appears on the medical records which may have caused misclassification and also did not provide a specific type of HF, such as HF with preserved ejection fraction (HFpEF) or HF with non-preserved ejection fraction (HFnpEF). Therefore, the association between obesity and a specific type of HF was not explored.

Additionally, as the data used in this study was collected from the Thailand DM/HT study, we did not have the opportunity to include physical activity and dietary behavior in the final model, which means that residual confounding may exist. We perform a sensitivity analysis and the evidence for causality from the E-values (Additional file 1: Table S2) looks relatively strong because substantial unmeasured confounding would be needed to reduce the observed association between obesity and new-onset HF. Despite these limitations, the study boasts several notable strengths. The large sample size analyzed in the study provides a robust dataset for our analyses. In addition, the study population is highly representative of Thai patients with HTN receiving continuous care nationwide, thereby enhancing the generalizability of our findings.

## Conclusion

In conclusion, our study indicates a positive association between elevated BMI and the incidence of new-onset HF in Thai patients with HTN. In particular, individuals with obesity exhibited a higher risk for new-onset HF as compared to those without obesity. The association between obesity and new-onset HF was attenuated among patients with uncontrolled BP; nevertheless, no heterogeneity was observed. Our findings highlight the importance of weight reduction in patients with HTN, irrespective of their BP control status, as a key strategy for mitigating the risk of HF development in the future.

### Supplementary Information

**Additional file 1**. Supplementary tables.

## Data Availability

Data cannot be shared publicly because the data set contains identifying information; additionally, the data belong to the Thailand DM/HT study of the Medical Research Network of the Consortium of Thai Medical Schools (MedResNet). Thus, ethical restrictions exist on the data set. Data are available from the Thai National Health Security Office (NHSO), Bangkok, Thailand (contact via https://dmht.thaimedresnet.org/) for researchers who meet the criteria for access to confidential data.

## Abbreviations

HF: Heart failure
BMI: Body mass index
HTN: Hypertension
BP: Blood pressure
SBP: Systolic blood pressure
DBP: Diastolic blood pressure
CVD: Cardiovascular disease
IHD: Ischemic heart disease
T2D: Type 2 diabetes
DLP: Dyslipidemia
