# Supplementary material for: Association between obesity and new-onset heart failure among patients with hypertension in Thailand
Source: J Health Popul Nutr. 2024 Feb 29;43:33. doi: 10.1186/s41043-024-00530-6 (PMC10905941; doi:10.1186/s41043-024-00530-6)
Supplement: Supplementary file 1 — Additional file 1. Supplementary tables. [file 41043_2024_530_MOESM1_ESM.docx]

**Appendix**

**Association between obesity and new-onset heart failure among patients with hypertension in Thailand**

*Boonsub Sakboonyarat^1^, Jaturon Poovieng^2^, *Ram Rangsin^1^*

^1^Department of Military and Community Medicine, Phramongkutklao College of Medicine, Bangkok 10400, Thailand

^2^Pulmonary and Critical Care Division, Department of Medicine, Phramongkutklao College of Medicine, Bangkok 10400, Thailand

***Corresponding author**

Ram Rangsin, MD, MPH, DrPH
Professor of Epidemiology

Department of Military and Community Medicine, Phramongkutklao College of Medicine, Bangkok 10400, Thailand

E-mail: r_rangsin@yahoo.com

Tel: +662354-7733

**Table S1.** Univariable log-binomial regression for factors associated with new-onset of heart failure among Thai patients with hypertension

| **Factors** | **Unadjusted Risk Ratio (95% CI)** | ***p*-value** |
| --- | --- | --- |
| **Sex** |  |  |
| Women | Ref. |  |
| Men | 0.95 (0.59-1.51) | 0.820 |
| **Age, years** | 1.04 (1.02-1.06) | <0.001 |
| **Regions** |  |  |
| North | Ref. |  |
| Central | 0.92 (0.51-1.68) | 0.796 |
| Northeast | 0.58 (0.29-1.16) | 0.122 |
| South | 1.38 (0.75-2.55) | 0.301 |
| **Location of outpatient clinics** | |  |
| Regional Hospital | Ref. |  |
| General Hospital | 0.77 (0.34-1.59) | 0.472 |
| Community Hospital | 0.34 (0.18-0.67) | 0.002 |
| Private Hospital | 0.96 (0.21-4.33) | 0.961 |
| Others | N/A | N/A |
| **Health insurance scheme** | |  |
| Universal health coverage | Ref. |  |
| Civil servant medical benefit | 0.58 (0.30-1.13) | 0.111 |
| Social security | 0.26 (0.04-1.88) | 0.183 |
| Others | N/A | N/A |
| **Smoking status** | |  |
| Never | Ref. |  |
| Ever smoker | 1.01 (0.54-1.87) | 0.983 |
| **Alcohol use** |  |  |
| Never | Ref. |  |
| Ever alcohol use | 1.24 (0.57-1.71) | 0.583 |
| **Diabetes comorbidity** | |  |
| No | Ref. |  |
| Yes | 0.95 (0.50-1.80) | 0.870 |
| **History of ischemic heart disease** | |  |
| No | Ref. |  |
| Yes | 4.36 (2.24-8.45) | <0.001 |
| **History of dyslipidemia** | |  |
| No | Ref. |  |
| Yes | 1.26 (0.77-2.04) | 0.355 |
| **History of renal insufficiency** | |  |
| No | Ref. |  |
| Yes | 3.13 (1.93-5.09) | <0.001 |
| **History of ACEI/ARB use** | |  |
| No | Ref. |  |
| Yes | 1.08 (0.68-1.73) | 0.743 |
| **Blood pressure control for the latest two consecutive visits** | |  |
| No | Ref. |  |
| Yes | 0.88 (0.56-1.38) | 0.565 |

CI: confidence interval

**Table S2.** Sensitivity analysis for unmeasured confounding using E-value for risk ratio.

| **Variables** | **Primary analysis** | **E-value for RR** | |
| --- | --- | --- | --- |
|  | **aRR (95% CI)*** | ***Point estimate*** | ***CI*** |
| **Body mass index (BMI)** |  |  |  |
| Continuous (linear) | 1.09 (1.05-1.14) | 1.40 | 1.28 |
| Continuous (quadratic) | 1.001 (1.001-1.002) | 1.03 | 1.03 |
| **BMI category, kg/m^2^** |  |  |  |
| <18.50 | 0.98 (0.36-2.69) | 1.17 | 1.00 |
| 18.50-22.99 | Ref. |  |  |
| 23.00-24.99 | 1.11 (0.50-2.47) | 1.46 | 1.00 |
| 25.00-29.99 | 1.57 (0.80-3.07) | 2.62 | 1.00 |
| ≥30.00 | 3.97 (1.95-8.10) | 7.40 | 3.31 |
| **BMI ≥ 25 kg/m^2^** |  |  |  |
| No | Ref. |  |  |
| Yes | 2.05 (1.24-3.39) | 3.52 | 1.79 |
| **BMI ≥ 30 kg/m^2^** |  |  |  |
| No |  |  |  |
| Yes | 3.19 (1.83-5.57) | 5.83 | 3.06 |

BMI: body mass index, aRR: adjusted Risk Ratio, 95% CI: 95% confidence interval

* Adjusting for age, sex, health schemes, geographic regions, location of outpatient clinic, type 2 diabetes, dyslipidemia, renal insufficiency, history of ischemic heart disease, smoking status, alcohol use, control blood pressure consecutively two latest visits, and ACEI/ARB use

**Example for E-value interpretation:**

Association between obesity and new-onset heart failure was observed, adjusted risk ratio 2.05 (95% CI: 1.24-3.39).

The E-value for the point estimate is 3.52.

This E-value can be interpreted as follows: “*The observed risk ratio of 2.05 could be explained away by an unmeasured confounder that was associated with both obesity and the heart failure by a risk ratio of 3.52-fold each, above and beyond the measured confounders, but weaker confounding could not do so*” [1]

The E-value for the lower confidence limit is 1.79, which can be interpreted as “*unmeasured confounders associated with obesity and heart failure by a risk ratio of 1.79-fold each could explain away the lower confidence limit, but weaker confounding could not*”.[1]

**References**

1. Linden A, Mathur MB, VanderWeele TJ. Conducting sensitivity analysis for unmeasured confounding in observational studies using E-values: The evalue package. Stata Journal. 2020;20.
